# New Appliances and Things Medical

**Published:** 1910-12-10

**Authors:** 


### NEW APPLIANCES & THINGS MEDICAL.

**[We shall be glad to receive at our Office, 28 & 29 Southampton Street, Strand, London, W.C., from the manufacturers, specimens of all new preparations and appliances.]**

### SPERMINOL.

(Leopold Stolkind and Co., Ltd., Moscow and Berlin.)

The Brown Sequard preparation and Poehl's spermin have been extensively tried, and the literature on the subject of these preparations is already voluminous. Sperminol, a new preparation which has been very extensively used in Russia, contains a larger percentage of spermin than Poehl's preparation. It is used both internally and as injections, and has been highly spoken of by Continental authorities in cases where the Brown Sequard preparation is indicated. In appearance it is a colourless, tasteless, and non-poisonous alcoholic solution of pure spermin, free from albuminates. In combination with iron extracts and ordinary simple tonics it is well worth a trial. The price is not stated.

